# Superdiversity, migration and use of internet-based health information – results of a cross-sectional survey conducted in 4 European countries

**DOI:** 10.1186/s12889-020-09329-6

**Published:** 2020-08-20

**Authors:** Florence Samkange-Zeeb, Liubov Borisova, Beatriz Padilla, Hannah Bradby, Jenny Phillimore, Hajo Zeeb, Tilman Brand

**Affiliations:** 1 Department of Prevention and Evaluation, Leibniz Institute for Prevention Research and Epidemiology – BIPS, Achterstr. 30, 28359 Bremen, Germany; 2 Department of Sociology, Uppsala University, Box 624, Se-751 26 Uppsala, Sweden; 3 Department of Sociology, University of South Florida, 42 E Fowler Ave, Tampa, FL 33620-5550 USA; 4 Instituto Universitario de Lisboa (ISCTE-IUL), Avenida das Forcas Armadas, 1649-026 Lisbon, Portugal; 5 School of Social Policy, Sociology and Criminology, University of Birmingham, Edgbaston, Birmingham, B15 2TT Great Britain; 6 Health Sciences Bremen, University of Bremen, Bibliothekstr.1, 28359 Bremen, Germany

**Keywords:** Internet-based health information, Migration, Digital divide

## Abstract

**Background:**

Studies of factors associated with the use of Internet-based health information generally focus on general, rather than migrant populations. This study looked into the reasons why Internet-based health information is used and the effects of migration-related factors, other socio-demographic characteristics and health-related factors on the tendency to consult the Internet.

**Methods:**

In a cross-sectional survey conducted in eight superdiverse neighbourhoods - two each in Birmingham, United Kingdom; Bremen, Germany; Lisbon, Portugal and Uppsala, Sweden - participants were presented with six scenarios and asked to indicate the resources they most relied on when addressing a health concern from a given list. The scenarios included establishing the underlying causes of a health concern and seeking information about prescription drugs, treatments and services available as part of the public healthcare system. The list of resources included the public healthcare system, alternative medicine, family and friends, and the Internet. Frequencies for which the Internet was consulted for each different scenario were calculated and compared across the participating cities. The association between consulting Internet-based health information and migration-related factors, and further socio-demographic characteristics as well as health-related factors such as self-reported health and health literacy was assessed using multivariable logistic regressions.

**Results:**

Of the 2570 participants from all four cities who were included in the analyses, 47% had a migrant background and 35% originated from non-EU countries. About a third reported relying on Internet-based health information for at least one of the given scenarios. The two most frequently chosen scenarios were to find out about other possible treatments and prescription drugs. Generally, using Internet-based health information was negatively associated with being a first generation migrant (OR 0.65; 95% CI 0.46–0.93), having poor local language competency (OR 0.25; 95% CI 0.14–0.45), older age (≥60 years, OR 0.21; 95% CI 0.15–0.31), low education (OR 0.35; 95% CI 0.24–0.50) and positively associated with low trust in physicians (OR 2.13; 95% CI 1.47–3.10).

**Conclusion:**

Our findings indicate the need to consider migration background and language competency when promoting the provision of healthcare services via the Internet so that information and services are widely accessible.

## Background

Over the past few years the Internet has evolved into a multidisciplinary tool affecting how societies function at different levels**.** According to Internet World Statistics [1], the proportion of the population who are Internet users worldwide went up from 20% in December 2007 to 55% in June 2018. In the European Union, it went up from 62% in 2007 to 89% in 2018 [2]. In 2014, 80% of respondents to a survey conducted in the EU28 countries reported having used the Internet for private purposes during the previous 12 months, 59% on a daily basis and 15% up to 3 times a week [3]. One area in which the Internet has become well-established is healthcare, in particular for sharing health-related information aimed at the general population. The easy availability of information has led to changes in how people seek access to healthcare, with more and more people getting health information from the Internet and not necessarily only from health professionals [4, 5]. Further, in some countries such as England, healthcare systems are increasingly offering online consultations to reduce unnecessary referrals and reduce pressure on outpatient appointments [6]. In the EU28 survey mentioned above, almost 60% of those who had used the Internet during the previous 12 months reported using it to search for health information. There is however considerable cross-country variation, with the highest use being observed in the Netherlands (73%), Sweden (70%) and Denmark (70%), and the lowest in Malta (49%) and Romania (47%) [3].

The kind of health information searched for on the Internet varies from general information on health topics such as how to improve one’s health, to information on specific conditions, diseases, treatment or medication [3, 4, 7]. To inform decision-making of service users, information on various healthcare services, including cost and quality comparisons, is made available on the Internet in some countries. However, while the Internet enhances easy distribution of information to the general population and potentially increases empowerment of individuals, some studies suggest that there is a divide in the use of digital health resources across population groups [8, 9]. Specifically, lower socioeconomic status, older age, and being male seem to be associated with lower use of digital health resources [10, 11]. The digital divide not only refers to limitations on Internet access, but also to limitations on whether information can be read, used and applied appropriately. Health literacy has been discussed as an important factor in explaining the use of digital resources across population groups [12].

While migrants consist of multiple heterogeneous groups regarding aspects such as reasons for migrating, education levels, residency status and socio-economic background, in health research they are largely classified as being disadvantaged compared to non-migrants. Many studies have observed that persons with a migrant background generally have poorer access to healthcare services compared to those without a migrant background. The reasons vary from language barriers, lack of information about available services, to structural issues such as little, or no access to healthcare services [13–16], as well as discrimination [17]. Recent developments in global migration, whereby unprecedented numbers of refugees, some speaking languages not previously encountered by healthcare providers in receiving countries, arrived in a relatively short space of time, have led to more attention being paid to the issue of access to, and provision of, good quality healthcare services for migrants and refugees [18–21]. The role the Internet can play in this regard has yet to be assessed. To date, the majority of studies in Europe that have looked at factors associated with Internet use for health information in the general population did not consider the influence of migration [3, 4, 7, 22].

The current study is based on a project that looked into how residents of superdiverse neighbourhoods sought to address their health concerns. Superdiverse neighbourhoods are areas populated by people originating from many different countries, who have different immigration statuses, socioeconomic backgrounds and who speak different languages, alongside local populations [23]. The project was carried out in four European cities: Birmingham, United Kingdom; Bremen, Germany; Lisbon, Portugal and Uppsala, Sweden [24]. In this context, participants were asked about the different resources they use when solving their health concerns, including the Internet.

This paper investigates the role of the Internet in the process of people addressing their health concerns. In their research on ethnic inequalities in access to e-health information in Israel, Mesch and colleagues [25, 26] tested the diversification hypothesis against the social stratification hypothesis. While the latter implies that patterns of Internet use for health information will mirror, and perhaps even broaden, inequalities observed in the society, the diversification hypothesis suggests that the Internet is a mode that can bridge and reduce inequalities regarding access to information and services. The authors found evidence to partly support both hypotheses, with the most disadvantaged reporting using the Internet for health information, but at the same time inequalities of access to e-health services being observed [26]. In our study we investigate the diversification versus the stratification hypothesis in the context of superdiverse neighbourhoods, which, due to their characteristics, may be expected to be conducive to diversification. To this end, we assess whether migrants, faced with language barriers, lack of adequate information on healthcare services and limited financial resources, rely on the Internet more than non-migrants. We compare the extent to which migrants rely on Internet-based health-related information compared to non-migrants, as well as looking at how socio-demographic characteristics, e.g. region of origin and socio-economic status, and health-related factors, such as self-rated health and health literacy are related to searching for health-related information on the Internet. Further, we also assess the purposes for which people relied on Internet-based health information.

## Methods

Between January and October 2017, a cross-sectional survey was conducted in two superdiverse neighbourhoods in each of four European cities: Birmingham, United Kingdom; Bremen, Germany; Lisbon, Portugal and Uppsala, Sweden. The neighbourhoods were selected on the basis of official data such as the population size, number of migrants and markers of social deprivation (see Supplementary data, Table 1). All the neighbourhoods that were selected have a heterogeneous population, comprising non-migrants as well as migrants originating from different countries and with a range of different migration statuses. The larger study adopted a mixed methods approach, in which the survey was one of the tools used to investigate the experiences and strategies of residents in superdiverse areas to address health concerns, examining the role of both formal and informal provision of healthcare services [24, 27]. In the first part of the study, qualitative interviews were conducted with residents and service providers from the eight selected neighbourhoods and the results formed the basis for the construction of the survey questionnaire.

### Recruitment of participants

Participants were recruited on a random basis, using the approach normally applied in similar population-based surveys in each country. The aim was to sample at least 300 persons from each neighbourhood; participants had to be at least 18 years old and reside in one of the neighbourhoods of interest. In Bremen, random address files of the respective neighbourhoods were obtained from the population office and prospective participants were contacted by letter. In the UK and Portugal, recruitment was done on a door-to-door basis and in Sweden via telephone calls. Further, in Germany a sub-sample of participants was recruited via respondent-driven sampling, a modified chain-referral sampling approach developed by Heckathorn [28].

All study material was translated into the languages most common in the respective neighbourhoods and the interviewer teams in all countries were multilingual. In Germany, Portugal and the UK the interviews were conducted face-to-face. In Sweden it was deemed culturally inappropriate to deploy a door-to-door sampling method, hence recruitment as well as the interviews were conducted over the phone. The telephone-based approach was also expected to increase the response rate. All participants provided informed consent, which was written for those interviewed face-to-face and verbal for those interviewed over the phone. Full ethical agreement for the study was received from the lead organisation, the University of Birmingham Ethical Review Committee, prior to the commencement of any research. The Ethical Review number is ERN_14–1111. Participants consented in writing for their data to be published in ways that ensure anonymity, thus identifying features have been removed.

Each partner organisation also received ethical approval from the respective authorities. These were the Ethics Committee University of Bremen, the ISCTE-IUL Ethical Review Committee as well as the Local health Authority in Lisbon (process number 8969/CES/2016) and the Swedish Ethical Committee (Etiknämnden) in Uppsala, (diarienummer 2015/112). Note; University of Bremen and ISCTE-IUL do not issue approval reference.

### Use of internet-based health information

Based on the results of the qualitative interviews, a set of six questions concerning the resources participants find most useful when looking for particular forms of information and support, as listed below, was constructed:

- Find out what their health concern is
- Information about prescription drugs
- Information about other possible treatments
- A recommendation for a specialist, hospital or other medical facility
- Emotional support in dealing with a health concern
- Practical advice for coping with day-to-day situations, e.g. pain, discomfort

For each scenario participants could choose one of the following responses:

i. Services provided by the public healthcare system/NHS
ii. Services paid for out of pocket
iii. Alternative or complementary medicine
iv. Services from another country
v. Family, friends, etc.
vi. Information from the Internet
vii. Other information sources (excluding the Internet)

Participants could also opt for ‘don’t know’ or refuse to give an answer. For each question, participants’ responses were coded ‘1’ if they had selected the Internet, and ‘0’ for all other responses. The outcome variable was then dichotomised as follows: 0 = not relying on Internet-based health information for any of the listed scenarios and 1 = relying on Internet-based health information for at least one of the listed scenarios.

### Socio-demographic characteristics and health-related factors

In addition to age, sex and socio-economic indicators, migration-related variables were also collected. The latter included participants’ place of birth, as well as that of their parents and grandparents, length of stay in the country of current residence and self-reported local language proficiency. Those born outside the country of current residence or whose parents or grandparents were not born in the country of residence were defined as having a migrant background. Self-reported local language proficiency was assessed on a five-point scale ranging from very good to very poor, which, for the analysis, was then dichotomized into high (very good and good) and low (medium to very poor). The International Standard Classification of Education (ISCED) was used to assess the level of education as follows: low: ISCED 0–2; medium: ISCED 3–4; and high: ISCED 5–6. Employment status was assessed as a yes/no variable.

The health-related factors assessed were self-reported health, health literacy, perceived discrimination in healthcare and trust in physicians. As is commonly done in population surveys [29], self-rated health was assessed based on the first question of the short form health survey questionnaire (SF-36) [30]. Participants rated their health on a 5-point scale from excellent to poor, and the responses were then dichotomized into good (excellent, very good, good) and poor (fair, poor).

To assess health literacy, participants were asked to rate how easy it is for them to find, understand, evaluate and use health information on a four-point scale, from 1 = very difficult to 4 = very easy. The assessment was based on the six-item short version of the European Health Literacy Survey Questionnaire (HLS-EU-Q6) [31]. The internal consistency was good (Cronbach’s alpha = 0.84). A mean score was then calculated for each participant and low health literacy was defined as a mean score between 1.0 and 2.0, according to Pelikan and colleagues [31].

For perceived discrimination, a question was adapted from the 2012 European Social Survey [32] to ask participants whether they would describe themselves as someone who is discriminated against by healthcare providers in the country of current residence (yes/no).

Finally, trust in physicians was assessed using a four-item short version of the trust in physicians scale [33] that has been used in the US-American General Social Survey [34]. Participants were asked to rate several statements about their general practitioners on a five-point scale (0 = strongly disagree to 4 = strongly agree), e.g. “I trust the doctor’s judgment about my medical care.” Participants’ responses were then summed up to a single score, ranging from 0 to 16. Internal consistency of the scale was acceptable (Cronbach’s alpha = 0.72). Values from 0 to 9 were classified as low trust in physicians, 10–12 as medium trust and 13–16 as high trust.

### Statistical analysis

The sample was weighted according to the age and sex distribution in the underlying population in each neighbourhood. Descriptive analysis of sample characteristics as well as the prevalence of purposes for which information from the Internet is generally found most useful when addressing a health concern was conducted using proportions with 95% confidence intervals (CI). Multivariable logistic regression analyses were carried out to assess factors associated with relying on health information from the Internet for the listed scenarios.

Several models were specified to assess the associations between migration-related variables, health-related variables, and relying on health information from the Internet. In the basic model (Model 1), socio-demographic factors, including age, employment status and migration background, were included. Migration specific factors (region of origin, local language competency and time living in the country) and health-related factors (self-rated health, health literacy, perceived discrimination, and trust in physicians) were then introduced in subsequent models (Models 2 and 3 respectively). For Model 2, collinearity was observed between the variable local language competency and time living in the country, hence the latter was excluded from the analysis. Uppsala was excluded from Model 3 as the variables of health literacy and trust in physicians were not collected in Sweden. To investigate the individual effects of each of the health-related variables, in Model 3 the respective variables were first added singly, and then combined.

Additional analyses were carried out to assess the consistency of the findings by using a different cut-off for the outcome variable (those rarely relying on health information from the Internet, (≤1 times) versus those frequently relying on health information from the Internet (≥2) and by conducting stratified analyses by survey location (city). All analyses were carried out using Stata 13 (StataCorp, College Station, Texas).

## Results

A total of 2692 persons took part in the survey and the proportion of participation ranged from 14% in Uppsala to 53% in Birmingham. One hundred and twenty-two persons were excluded from the analysis as they had missing data for one or more of the core variables such as age, education, migration background and self-rated health. The majority of participants included in the analysis were younger than 45 years (53%) and female (51%) (Table 1). Almost a third of participants were first generation migrants (29.3%), while slightly less than a fifth were descendants of migrants (17.9%). The majority of persons with a migrant background originated from non-EU countries, and at the time of the survey, more than 38% of them had been living in the country of current residence for up to ten years. Further, more than two thirds rated their local language competency as good/very good.

**Table 1:** Weighted sample characteristics and proportion of participants across all four cities stratified according to relying on Internet-based health information for at least one of the given scenarios

	Sample characteristics (%)	Internet-based health information mostly relied on (%) (95%-CI)	
		No	Yes
Total (*n* = 2570)	100	65.5 (63.2–67.6)	34.5 (32.4–36.8)
City, Country			
Birmingham, UK	20.4	66.4 (62.0–70.6)	33.6 (29.4–38.0)
Bremen, Germany	33.5	53.6 (49.8–57.3)	46.4 (42.7–50.2)
Lisbon, Portugal	22.6	91.8 (89.7–93.9)	8.2 (6.1–10.9)
Uppsala, Sweden	23.6	56.3 (50.4–61.9)	43.7 (38.1–49.6)
Age groups in years			
18–29	26.9	55.0 (49.5–60.4)	45.0 (39.6–50.5)
40–44	25.8	57.2 (52.7–61.6)	42.8 (38.4–47.3)
45–59	21.5	71.2 (67.2–75.0)	28.8 (25.0–32.8)
≥ 60	25.8	79.8 (76.9–82.4)	20.2 (17.6–23.1)
Gender			
Women	51.1	65.1 (62.2–67.9)	34.9 (32.1–37.8)
Men	48.9	65.8 (62.4–69.1)	34.2 (30.9–37.6)
Education			
Low	34.8	86.2 (82.7–89.0)	13.8 (11.0–17.3)
Medium	33.3	57.9 (53.9–61.8)	42.1 (38.2–46.1)
High	31.9	50.8 (47.0–54.6)	49.2 (45.4–53.0)
Unemployed			
Yes	9.3	77.1 (70.1–82.8)	22.9 (17.2–29.9)
No	90.7	64.3 (61.9–66.6)	35.7 (33.4–38.1)
Migration background			
None	52.8	64.9 (62.0–67.7)	35.1 (32.3–38.0)
Migrant	29.3	74.3 (70.5–77.7)	25.7 (22.3–29.5)
Descendants of migrants	17.9	52.7 (46.3–58.9)	47.3 (41.1–53.7)
Region of origin			
No migration background	52.8	64.9 (62.0–67.8)	35.1 (32.2–38.0)
EU-15	6.3	60.3 (50.1–69.7)	39.7 (30.3–49.9)
EU-28	5.5	54.8 (45.6–63.7)	45.2 (36.3–54.5)
Non-EU	35.4	68.8 (64.8–72.5)	31.2 (27.5–35.2)
Years living in the country^a^			
0–10	38.1	74.0 (67.5–79.6)	26.0 (20.4–32.5)
11–20	24.6	72.8 (63.7–80.3)	27.2 (19.7–36.3)
> 20	37.3	76.5 (70.5–81.7)	23.5 (18.3–29.5)
Local language competency^a^			
Poor/fair	31.9	89.9 (84.6–93.6)	10.1 (6.4–15.4)
Good/very good	68.1	66.8 (61.8–71.5)	33.2 (28.5–38.2)
Health literacy^b^			
Low	11.9	77.0 (70.1–82.8)	23.0 (17.2–29.9)
Medium/high	88.1	66.6 (64.3–69.3)	33.1 (30.7–35.7)
Self-rated health			
Poor	22.5	81.6 (77.9–84.8)	18.4 (15.2–22.1)
Good	77.5	60.8 (58.2–63.4)	39.2 (36.6–41.8)
Trust in physicians^b^			
Low	25.6	53.8 (48.9–58.7)	46.2 (41.3–51.1)
Medium	44.8	67.6 (64.0–71.1)	32.4 (28.9–36.0)
High	29.6	80.2 (76.2–83.8)	19.8 (16.3–23.8)
Perceived discrimination			
No	93.2	66.1 (63.8–68.4)	33.9 (31.6–36.2)
Yes	6.8	56.4 (48.2–64.3)	43.6 (35.7–51.8)

Note: Sample characteristics were weighted by the age and gender distribution of the underlying population

^a^ assessed only among migrants

^b^not assessed in Sweden

Differences between cities were observed for the proportion of women, age distribution, proportions of persons with a low educational level, as well as those with a migrant background. Regarding the last two variables, more than two-thirds of the participants in Lisbon had a low level of education, compared to less than a fifth in Uppsala and Bremen, while Birmingham had the highest proportion of persons with a migrant background (78%), followed by Uppsala with 42%. Further, almost all participants from Lisbon who had a migrant background originated from non-EU countries (see Supplementary data, Table 2 and Table 3).

### Relying on internet-based health information when addressing a health concern

**Fig. 1:**
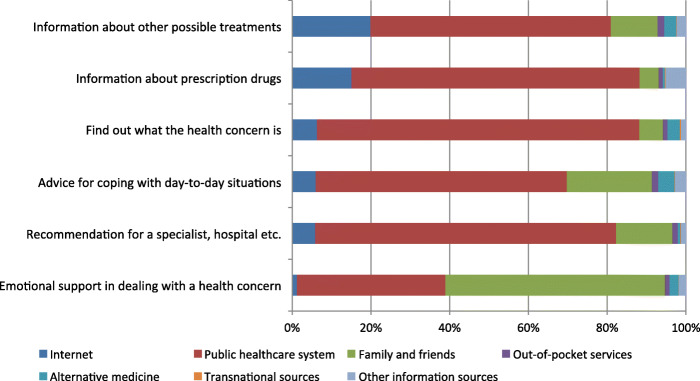
Distribution of resources participants mostly relied on for the given scenarios

In general, the participants mostly relied on the public healthcare system for each of the given scenarios apart from “emotional support in dealing with a health concern”. For the latter, the resource mostly relied on was family and friends (Fig. 1).

About a third of the participants in the four cities reported relying on the Internet for at least one of the given scenarios when addressing a health concern (Table 1). Internet-based health information was mostly relied on “to find out about other treatments”, followed by “to find out about prescription drugs”. Only a small minority reported relying on the Internet “to find emotional support in dealing with a health concern” (Fig. 1). First generation migrants reported relying on the Internet less than non-migrants and descendants of migrants, with the highest proportion being observed for the latter (47%). Further, European migrants reported relying on the Internet more often than non-EU migrants and non-migrants. While differences could be observed between the cities, with the proportion of those relying on health information from the Internet was highest in Bremen and Uppsala (in total more than 40% of participants in each city) and lowest in Portugal (less than 10%), the proportion generally decreased with age and was lower among men, the less educated, the unemployed, those with low local language competency, with low health literacy, as well as those with poor self-reported health. Regarding trust in physicians and perceived discrimination, the proportion of participants relying on the Internet was higher among those reporting low trust in physicians and perceived discrimination.

### Factors associated with relying on health information from the internet when addressing a health concern

Regression analysis results partly confirmed observations made based on descriptive statistics (Table 2). Compared to non-migrants, being a first generation migrant was significantly associated with reduced odds of relying on the Internet (OR 0.68, 95% CI 0.50–0.93) (Model 1). While the prevalence for descendants of migrants was slightly higher than that of non-migrants: the finding was not statistically significant. Considering migration-related factors combined (Model 2), region of origin was not significantly associated with relying on health information from the Internet. Local language competency however appeared to be associated with relying on the Internet for health information, with those reporting poor language competency relying less on the Internet compared to non-migrants (OR 0.25, 95% CI 0.14–0.45).

**Table 2:** Socio-demographic, migration-related and health-related factors associated with relying on the Internet-based information when addressing health concerns (multivariable logistic regression)

Variables	Model 1 OR (95% CI)	Model 2 OR (95% CI)	Model 3^**#**^ OR (95% CI)
**Migration background** (Ref. no migration background)			
*Migrants*	0.68 (0.50–0.93)*	–	0.65 (0.46–0.93)*
*Descendants of migrants*	1.02 (0.74–1.41)	–	1.00 (0.71–1.41)
**City, country** (Ref. Birmingham, UK)			
Bremen, DE	1.44 (1.06–1.94)*	1.50 (1.10–2.06)*	1.36 (0.99–1.87)
Lisbon, PT	0.21 (0.14–0.32)*	0.23 (0.15–0.34)*	0.22 (0.15–0.34)*
Uppsala, SW	1.27 (0.88–1.85)	1. 28 (0.87–1. 88)	–
**Age in years** (Ref. 18–29)			
30–44	0.97 (0.70–1.34)	1.03 (0.74–1.43)	0.69 (0.48–0,.99)*
45–59	0.46 (0.33–0.64)*	0.46 (0.33–0.65)*	0.37 (0.25–0.53)*
≥ 60	0.29 (0.21–0.40)*	0.29 (0.21–0.40)*	0.21 (0.15–0.31)*
**Gender** (Ref. Women)			
*Men*	0.85 (0.67–1.06)	0.86 (0.68–1.08)	0.79 (0.62–1.03)
**Education** (Ref. High)			
*Low*	0.29 (0.20–0.43)*	0.31 (0.22–0.45)*	0.35 (0.24–0.50)*
*Medium*	0.68 (0.53–0.87)*	0.67 (0.52–0.86)*	0.72 (0.54–0.97)*
**Unemployed** (Ref. No)			
Yes	0.69 (0.46–1.04)	0.69 (0.45–1.03)	0.68 (0.43–1.07)
**Region of origin** (Ref. No migration background)			
*EU-15*	–	0.84 (0.51–1.39)	–
*EU-27*	–	1.10 (0.67–1.79)	–
*Non-EU*	–	1.09 (0.74–1.59)	–
**Local language competency** (Ref. No migration background)			
*Good*	–	0.86 (0.57–1.28)	–
*Poor*	–	0.25 (0.14–0.45)*	–
**Self-rated health** (Ref. Good)			
*Poor*	–	–	0.84 (0.59–1.20)
**Health literacy** (Ref. High)			
*Low*	–	–	0.87 (0.55–1.38)
**Trust in physicians (**Ref. High)			
*Low*	–	–	2.13 (1.47–3.10)*
*Medium*	–	–	1.66 (1.18–2.32)*
**Perceived discrimination (**Ref. No)			
*Yes*	–	–	1.09 (0.67–1.76)

Note: Sample characteristics were weighted by the age and gender distribution of the underlying population

*p < 0.05

^#^Sweden excluded from model 3 as information on health literacy and trust in physicians was not collected

Regarding city being surveyed, in all three models the reported reliance on the Internet was significantly lower in Lisbon compared to Birmingham. In Bremen it was higher than in Birmingham, but significant only in Models 1 and 2. The increased odds ratios of using Internet-based health information in Uppsala were higher than in Birmingham but not significant in any of the models.

Use of the Internet decreased with age, with those aged 60 and above having more than 70% reduced odds compared to those younger than 30 years of age. Those with lower levels of education also had significantly reduced odds of relying on the Internet, compared to those with higher levels of education (Table 2). Compared to women, men tended to rely less on the Internet. These results were however not statistically significant. Being unemployed was also not significantly associated with relying on the Internet.

The results of the third model, in which health-related factors were added to the basic model, show a tendency for poor self-rated health and low health literacy to reduce reliance on Internet-based health information, albeit not significantly. Lack of trust in physicians on the other hand increased the odds of relying on the Internet, with the odds among participants with low trust doubling compared with those with high trust (OR 2.17, 95% CI 1.50–3.14). Perceived discrimination increased the odds of relying more on the Internet slightly by 6%, but the result was not statistically significant. The findings for trust in physicians did not change when the factor was added to the model individually or combined with the other health-related factors. Slight changes in the value of the odds ratios were observed for self-rated heath, health literacy and perceived discrimination. The association between each of the factors and relying on the Internet was stronger when each was added individually to the basic model than in the combined model. The direction of the findings however did not change, and neither were the findings statistically significant. Adding language competency to the model did not change any of the findings.

### Additional analyses

Using a different cut-off for relying on the Internet (≤1 times vs. ≥2) did not change the general pattern of the results. Although being a first generation migrant was observed to be associated with reduced odds of relying on Internet-based health information compared to non-migrants, as in the main analysis, the results in this instance were not statistically significant. Increasing age and having low education were on the other hand significantly associated with lower odds of relying on the Internet, while low trust in physicians was associated with higher odds of doing so (see Supplementary data, Table 4).

Stratifying the analysis by survey city revealed some differences between the four sites. While being a first generation migrant was associated with reduced odds of relying on the Internet for health information across all cities, the effect was statistically significant only in Lisbon (see Supplementary data, Tables 5–8). Similarly, being unemployed was significantly associated with reduced odds of relying on the Internet only in Lisbon (OR 0.17, 95% CI 0.03–0.86). Although the findings were not statistically significant, perceived discrimination and health literacy also appear to have had a stronger impact in Lisbon than in Birmingham and Bremen. In Lisbon, the odds of those perceiving discrimination relying on the Internet were more than double of those not perceiving discrimination, while the odds of persons with low health literacy relying on the Internet were reduced by 41% compared to those with high health literacy. In both instances migration background does not seem to have played a significant role. In comparison, the odds ratios for both variables in Birmingham and Bremen were close to 1.

Trust in physicians on the other hand appears to have played a more important role in Bremen than in Lisbon and Birmingham, with the odds of those reporting low and medium trust being significantly higher than of those reporting high trust. Other than in the combined analysis and in the other cities, in Uppsala men tended to report relying on the Internet more often than women. The findings were however not statistically significant.

## Discussion

Based on a survey conducted in eight superdiverse neighbourhoods in four European cities, this study looked at how migration background and other migration-related factors, along with health-related and socio-economic factors, might influence the use of Internet-based health-related information when addressing health concerns. In general, being a first generation migrant, having poor local language competency, being older and having a low level of education were associated with reduced odds of relying on Internet-based health information for the scenarios presented to the participants, while having low/medium trust in physicians was associated with increased odds of doing so.

The findings regarding migrants are particularly interesting as they are in contradiction to what we would have expected based on the literature [13–17]. The research suggests that migrants are disadvantaged in their new countries for reasons that include language barriers, poorer living conditions and limited access to services and information. We therefore expected to find results similar to those reported by Mesch and colleagues [25, 26], whereby, in support of the diversification hypothesis, migrants relied on Internet-based health information more than non-migrants. We further expected that migrants would access information on the Internet in their native language, as a way to overcome the language barrier. As already reported, migration-related factors were not associated with an *increased* reliance on health information from the Internet. Indeed, being a first generation migrant and having poor local language competency was associated with significantly *reduced* odds of relying on Internet-based health information for the given scenarios. While this finding does not support our initial expectation, an explanation for this could be related to the type of information migrants can access online in their native language and its appropriateness for local use. Whereas general information on health and illness is likely to be easily available in a broad spectrum of languages on the Internet, getting Internet-based information about *local* health care services and details regarding diagnosis, treatment and medication in the country of residence is bound to be difficult without some fluency in the local language.

A further explanation could be that participants were not aware of the availability of information on the Internet, or that the information they needed was mainly available in the local language and not in their own language [26, 35]. Reliance on social support structures, i.e. personal contact, could also play a role, especially when one does not speak the local language. This aspect came up in several interviews during the qualitative part of this study, whereby those with language problems reported relying on the help of family members, friends, acquaintances as well as non-governmental organisations when trying to access health services [24]. In Portugal for instance, non-governmental organisations play a big role in supporting migrants to access appropriate health services, which migrants cannot manage alone due to language difficulties, unfamiliarity with the health system or lack of legal documentation [36]. This might indeed explain the particularly low reliance on Internet-based health information that was observed in Lisbon.

Another factor that proved to have an effect on searching for health-related information on the Internet was trust in physicians. Our findings in this regard, whereby low trust in physicians was associated with higher odds of using health information from the Internet, are in line with the existing literature [4]. Several studies have shown that a lack of trust in physicians, respectively medical personnel, leads to a reluctance to use health services. For instance, Phillimore and colleagues [24] show that lack of trust was associated with a range of different healthcare seeking strategies, including use of the Internet, using non-NHS resources and relying heavily on social networks for support.

Our findings add to the discussion of the digital divide, which manifests in several spheres. First, low *education* level was observed to be associated with reduced odds for relying on health information from the internet. Research has shown that certain digital competencies linked directly to education are needed to be able to use the Internet to search for information. This introduces inequalities in access to and use of information, with those with lower levels of education facing more challenges compared to those with more education [37–42].

Second, our findings also indicate the presence of a geographical digital divide within Europe. As the survey was carried out in four different countries, we were able to observe strong cross-country differences in line with other research on Europe [43]. Indeed, similar to our findings, results of the European citizens’ digital health literacy survey conducted in 2014 [3] suggest that the proportion of participants reporting *not* having used the Internet to search for health-related information 12 months prior to the survey was higher in Portugal (50%), compared to Germany (42%), the UK (40%) and Sweden (29%).

Our results regarding reduced use of Internet-based health information with increasing age support those of other research on age-related digital divide [44]. Interestingly though, the effect of age, which was significant in the combined analysis, completely disappears in Uppsala, but is present in the other cities. This opens up a new potential for research in age-related digital divide differences across Europe.

Finally, the suggestion that migration-related factors support the digital divide is a finding which merits greater attention. The migration-related aspects of the digital divide is underexplored in the field of health, but supported by research on the use of electronic services in the public sector [45].

### Strengths and limitations

A main strength of this paper is that it looked at how migration background, along with health-related and socio-economic factors, might have an effect on using health-related information on the Internet, something which has not been addressed in previous research [3, 4, 7, 22]. The fact that the survey was carried out in four cities in four different countries is a further strength, in that it allows cross-country comparisons. The use of different recruitment methods in the different settings is a design weakness since differential selection bias in the different countries may have compromised comparability to some extent. This, for instance, could have led to the low participation proportion observed in Uppsala, where recruitment was done by telephone, compared to Birmingham, where it was done door-to-door. To counteract this potential bias, we weighted the samples by the age and sex distributions in the underlying population of the neighbourhoods. After weighting, the distributions in our sample were very similar to the distributions in the neighbourhoods, not only for age and sex, but also with regard to other characteristics such as proportion of migrants.

Regarding our findings, we cannot tease out whether general affinity for Internet use is the driving factor for the observations we made, from the possibility that the availability and quality of the health information is the driving factor. Further, although the scenarios presented in our questionnaire mirror issues commonly found in studies of the use of the Internet for health information [3, 11, 46], we cannot rule out that there are other aspects for which the participants rely on the Internet which we did not cover. Hence, future studies could look into why some population groups rely more on the Internet while others do not. A concerted effort should be made to find out the needs of the potential users as well as how reliable, multilingual health information can be made more readily and easily available on the Internet. Finally, as we did not collect information on migrants’ previous experiences of using the Internet in their country of origin or how they access digital resources in their current place of residence, we cannot assess how these factors might have affected their use of the Internet for health information. Future studies should take these aspects into consideration.

## Conclusion

The digitalisation of health services will continue to expand in the future. Some services, for example in the UK and Portugal, already use the Internet for booking appointments, with those accessing services via the Internet getting priority. In spite of the proposed positive aspects of placing health services and information online, the risk of leaving some population groups behind should not be underestimated. Our results indicate that the digital divide exists not only between generations and socioeconomic strata, but also between migrants and non-migrants. Providing health information in different languages might help close this gap, but an inclusive internet-health strategy needs to include enhancing the capacity and IT competency of newcomers to be able to make use of the Internet in their new country of residence, in order to avoid amplifying a digital divide around migration background.

## Supplementary information

**Additional file 1 Supplementary Table 1**: Characteristics of the comparison countries and neighbourhoods. **Supplementary Table 2**: Further breakdown of the regions of origin of migrants, stratified by city. **Supplementary Table 3**: Weighted sample characteristics stratified by city . **Supplementary Table 4**: Socio-demographic, migration-related and health-related factors associated with use of the Internet when addressing health concerns (multivariable logistic regression) (less than twice users versus at least twice users) . **Supplementary Table 5**: Socio-demographic, migration-related and health-related factors associated with relying on the Internet for information when addressing health concerns (multivariable logistic regression): Birmingham, UK (*n* = 524) . **Supplementary Table 6**: Socio-demographic, migration-related and health-related factors associated with relying on the Internet for information when addressing. Health concerns (multivariable logistic regression): Bremen, Germany (*n* = 841). **Supplementary Table 7**: Socio-demographic, migration-related and health-related factors associated with relying on the Internet for information when addressing health concerns (multivariable logistic regression): Lisbon, Portugal (*n* = 572). **Supplementary Table 8**: Socio-demographic, migration-related and health-related factors associated with relying on the Internet for information when addressing health concerns (multivariable logistic regression): Uppsala, Sweden (*n* = 571)

## Data Availability

The data supporting the conclusions of this article are available from the corresponding author, HZ, upon reasonable request.

## Abbreviations

CI: Confidence Intervals
EU: European Union
ISCED: International Standard Classification of Education
NHS: National Health Service
SF-36: Short Form Health Survey
OR: Odds Ratio
UK: United Kingdom
HLS-EU-Q6: European Health Literacy Survey Questionnaire (6-item Version)
