# Supplementary material for: Superdiversity, migration and use of internet-based health information – results of a cross-sectional survey conducted in 4 European countries
Source: BMC Public Health. 2020 Aug 20;20:1263. doi: 10.1186/s12889-020-09329-6 (PMC7439663; doi:10.1186/s12889-020-09329-6)
Supplement: Supplementary file 1 — Additional file 1 Supplementary Table 1: Characteristics of the comparison countries and neighbourhoods. Supplementary Table 2: Further breakdown of the regions of origin of migrants, stratified by city. Supplementary Table 3: Weighted sample characteristics stratified by city . Supplementary Table 4: Socio-demographic, migration-related and health-related factors associated with use of the Internet when addressing health concerns (multivariable logistic regression) (less than twice users versus at least twice users) . Supplementary Table 5: Socio-demographic, migration-related and health-related factors associated with relying on the Internet for information when addressing health concerns (multivariable logistic regression): Birmingham, UK (n = 524) . Supplementary Table 6: Socio-demographic, migration-related and health-related factors associated with relying on the Internet for information when addressing. Health concerns (multivariable logistic regression): Bremen, Germany (n = 841). Supplementary Table 7: Socio-demographic, migration-related and health-related factors associated with relying on the Internet for information when addressing health concerns (multivariable logistic regression): Lisbon, Portugal (n = 572). Supplementary Table 8: Socio-demographic, migration-related and health-related factors associated with relying on the Internet for information when addressing health concerns (multivariable logistic regression): Uppsala, Sweden (n = 571) [file 12889_2020_9329_MOESM1_ESM.docx]

Supplementary Table 1: Characteristics of the comparison countries and neighbourhoods

|  | Health and welfare regimes | City and neighbourhoods |
| --- | --- | --- |
| Germany | Conservative welfare regime  Universal, corporatist health care system, decentralized and self-governing. Compulsory health insurance based on income covers 85% of the population. Direct access to services with choice of provider. Migrants receive a health insurance card allowing access to medical help for acute illness, pain and pregnancy. Without insurance, people must pay or use volunteer doctors, CSOs and welfare organizations. There is no functioning interpretation system. The healthcare ecosystem is very complex so people struggle to understand entitlements. The ecosystem has been transformed into a competitive health market with statutory health insurers behaving as competing corporations. Medical professionals are supposed to report irregular migrants to immigration authorities. | Bremen: 10^th^ largest city  554646 residents, 30% people from migrant background (deprived and skilled) from 162 countries.  Gröpelingen: 35,055 residents, 44.1 % PMB, 2^nd^ highest number welfare dependants (33.3 %), high deprivation. Long history migration. Increasing welfare dependency.  Neustadt: 43,699 residents, 26 % PMB, students, migrants and middle-class. Decreasing welfare dependency with early gentrification. Long history migration. |
| Portugal | Southern European welfare regime  Health system comprised of multiple sectors including a universal national health service (NHS) with co-payment scheme and exemptions for certain populations. Health subsystems include health insurance for public servants, a growing private insurance health sector and the lottery funded charity-led parallel health service of Santa Casa da Misericordia (SCML) for vulnerable populations. The economic crisis affected provision and quality of health services as TROIKA imposed severe. Most irregular migrants’ exemptions were removed making access problematic. NHS professionals cannot report irregular migrants to authorities due to professional ethics. | Lisbon: capital & largest city  547733 residents, housing migrants from 172 countries, recent arrival of refugees.  Lumiar: 25,000 residents, 15 % migrants, high welfare dependency, high deprivation.  Mouraria: 15,000 residents, migrants from 30 countries since the 1970s. Welfare dependency paired with gentrification. |
| Sweden | Social Democratic welfare regime  Comprehensive universal system. Equity is prioritised through redistributive policies in the form of statutory and municipal taxes, benefits and services aimed at mitigating the damaging effects of poverty. The system of fiscal and non-fiscal universal benefits, distributed with little means-testing imply extensive public-sector employment in health and social care. Healthcare and welfare available to whole population for a small fee. Only immigrants with legal rights of domicile can access non-urgent care. Very limited private sector. Provision through for-profit corporations increasing. Limited austerity since Sweden’s major financial crisis and contraction of the welfare state occurred in the 1990s. Emphasis on individual responsibility, healthy living and active lifestyles. | Uppsala: 4th largest city.  202625 residents, people from migrant background from 174 countries (deprived and skilled).  Gottsunda: 9,924 residents, 53 % PMB, high welfare dependency. Long history migration. Significant municipal investment addressing social problems.  Sävja: 5,330 residents, 39 % PMB, pockets of deprivation and affluence. Few municipal resources.  Occasional social unrest. |
| UK | Liberal welfare regime  The UK's NHS introduced as a universal system with primary and secondary healthcare free to all. The past 20 years have seen constant attempts at restructuring to slow down spiraling costs. Shortages of doctors and nurses with the system said to be in crisis and Government refusing to increase the budget. Restructuring in 2013 introduced service commissioning to introduce competition, reduce costs and offer choice for health “consumers”. Widespread concerns about capacity to meet rising demand, the exacerbation of recruitment difficulties, reduced investment, long-term under-funding of mental health provision and cuts in public health and social care budgets. Immigration legislation denies undocumented migrants and failed asylum seekers free access beyond emergency care. NHS workers are expected to report and refuse to treat undocumented migrants. | Birmingham: 2^nd^ largest city.  1073045 residents, 22% foreign born, 47% ethnic minorities from 187 countries.  Lozells and East Handsworth: 31,074 residents, 44.9 % FB, 89.2 % EM, 5^th^ most deprived ward. Long history migration with recent increases and diversification.  Edgbaston: 24,426 residents, 29.2 % FB, 42.2 % EM, 34^th^ most deprived ward. More recent history migration. |

Data for Germany: 2012 national census and Arbeitnehmerkammer: Bericht zur sozialen Lage 2013

Data for Portugal: migrant definition: foreign born and ethnic minorities

Data for Sweden: foreign born and ethnic minorities

Data for the UK: 2011 Census

Supplementary Table 2: Further breakdown of the regions of origin of migrants, stratified by city

|  | UK  (n=517) | Germany  (n=839) | Portugal  (n=572) | Sweden  (n=571) | Total  (n=2499) |
| --- | --- | --- | --- | --- | --- |
| Region of origin |  |  |  |  |  |
| No migrant background | 21.92 | 66.02 | 62.11 | 58.55 | 54.37 |
| Western Europe | 7.19 | 4.99 | 1.90 | 13.32 | 6.74 |
| Eastern Europe and Russia | 4.29 | 9.67 | 0.77 | 6.30 | 5.75 |
| Southeast Europe | 3.24 | 8.76 | 0.70 | 3.02 | 4.43 |
| North Africa and Middle East | 16.84 | 3.23 | 1.69 | 10.06 | 7.29 |
| Sub-Saharan Africa | 10.65 | 4.29 | 18.19 | 2.10 | 8.20 |
| Central and East Asia | 2.89 | 0.29 | 3.47 | 2.17 | 1.99 |
| Southeast Asia | 18.71 | 0.96 | 7.18 | 0.83 | 5.94 |
| Latin America | 0.34 | 1.22 | 3.83 | 3.08 | 2.08 |
| Caribbean | 12.74 | 0.37 | 0.00 | 0.23 | 2.77 |
| Other | 1.20 | 0.21 | 0.17 | 0.34 | 0.43 |

Supplementary Table 3: Weighted sample characteristics stratified by city

|  | Birmingham, UK  (n=525) | Bremen, Germany  (n=841) | Lisbon, Portugal  (n=572) | Uppsala, Sweden  (n=571) |
| --- | --- | --- | --- | --- |
| Age groups in years | | | | |
| 18-29 | 38.8 | 24.8 | 17.8 | 28.7 |
| 30-44 | 25.8 | 26.2 | 27.6 | 21.5 |
| 45-59 | 18.3 | 23.9 | 23.1 | 20.3 |
| ≥60 | 17.0 | 25.0 | 31.5 | 29.5 |
| Gender | | | | |
| Women | 50.8 | 49.4 | 53.5 | 51.7 |
| Men | 49.2 | 50.7 | 46.5 | 46.5 |
| Education | | | | |
| Low | 43.5 | 15.8 | 70.6 | 19.0 |
| Medium | 32.4 | 47.0 | 15.8 | 33.8 |
| High | 24.1 | 37.2 | 13.6 | 47.3 |
| Unemployed | | | | |
| Yes | 10.3 | 10.2 | 12.3 | 4.2 |
| No | 89.7 | 89.8 | 87.7 | 95.8 |
| Migration background | | | | |
| None | 21.4 | 65.8 | 62.1 | 58.6 |
| Migrant | 50.7 | 13.4 | 33.8 | 21.4 |
| Descendants of migrants | 27.9 | 20.8 | 4.1 | 20.1 |
| Region of origin | | | | |
| No migration background | 21.6 | 65.8 | 62.1 | 58.6 |
| EU-15 | 6.9 | 4.5 | 1.3 | 11.9 |
| EU-28 | 5.6 | 9.2 | 1.0 | 3.5 |
| Non-EU | 65.9 | 20.4 | 35.6 | 26.1 |
| Years living in the country* | | | | |
| 0-10 | 42.0 | 43.5 | 42.7 | 22.1 |
| 11-20 | 23.2 | 19.8 | 24.6 | 29.8 |
| >20 | 34.9 | 36.7 | 32.7 | 48.1 |
| Local language competency* | | | | |
| Poor/fair | 30.7 | 43.1 | 45.5 | 10.4 |
| Good/very good | 69.3 | 56.9 | 54.5 | 89.6 |
| Self-rated health | | | | |
| Poor | 18.3 | 15.4 | 38.4 | 21.9 |
| Good | 81.7 | 84.7 | 61.6 | 78.1 |
| Health literacy** |  |  |  |  |
| Low | 8.4 | 10.8 | 17.0 | - |
| Medium/high | 91.6 | 89.2 | 83.0 | - |
| Trust in physicians** | | | | |
| Low | 19.9 | 41.0 | 8.4 | - |
| Medium | 42.6 | 44.1 | 48.2 | - |
| High | 37.6 | 15.0 | 43.4 | - |
| Perceived discrimination | | | | |
| No | 93.9 | 91.3 | 95.0 | 93.4 |
| Yes | 6.1 | 8.7 | 5.0 | 6.6 |

Note: Sample characteristics were weighted by the age and gender distribution of the underlying population

* assessed only among migrants

**not assessed in Sweden

Supplementary Table 4: Socio-demographic, migration-related and health-related factors associated with use of the Internet when addressing health concerns (multivariable logistic regression) (less than twice users versus at least twice users)

| **Variables** | | **Model 1**  **OR (95% CI)** | | **Model 2**  **OR (95% CI)** | | **Model 3^#^**  **OR (95% CI)** |
| --- | --- | --- | --- | --- | --- | --- |
| **Migration background** (Ref. no migration background) | | | | | | |
| *Migrants* | | 0.85 (0.58-1.25) | | - | | 0.80 (0.50-1.27) |
| *Descendants of migrants* | | 0.81 (0.57-1.16) | | - | | 0.90 (0.60-1.36) |
| **City, country** (Ref. Birmingham, UK) | | | | | | |
| *Bremen, Germany* | | 1.04 (0.72-1.51) | | 1.07 (0.73-1.56) | | 1.02 (0.69-1.49) |
| *Lisbon, Portugal* | | 0.23 (0.13-0.40)* | | 0.23 (0.13-0.41)* | | 0.26 (0.15-0.47)* |
| *Uppsala, Sweden* | | 1.07 (0.72-1.59) | | 1. 07 (0.71-1. 59) | | - |
| **Age in years** (Ref. 18-29) | |  | |  | |  |
| *30-44* | | 0.90 (0.63-1.29) | | 0.93 (0.65-1.33) | | 0.58 (0.38-0.90)* |
| *45-59* | | 0.57 (0.39-0.82)* | | 0.57 (0.39-0.83)* | | 0.36 (0.24-0.55)* |
| *≥60* | | 0.31 (0.21-0.45)* | | 0.31 (0.21-0.46)* | | 0.25 (0.16-0.40)* |
| **Gender** (Ref. Women) | | | | | | |
| *Men* | | 0.82 (0.63-1.06) | | 0.82 (0.63-1.07) | | 0.80 (0.58-1.10) |
| **Education** (Ref. High) | | | | | | |
| *Low* | | 0.31 (0.20-0.48)* | | 0.33 (0.21-0.52)* | | 0.41 (0.24-0.68)* |
| *Medium* | | 0.74 (0.55-1.00) | | 0.74 (0.55-0.99)* | | 0.98 (0.68-1.41) |
| **Unemployed** (Ref. no) | | | | | | |
| *Yes* | | 1.02 (0.61-1.73) | | 1.03 (0.61-1.74) | | 1.29 (0.73-2.30) |
| **Region of origin** (Ref. no migration background) | | | | | | |
| *EU-15* | | - | | 0.77 (0.43-1.38) | | - |
| *EU-27* | | - | | 0.85 (0.47-1.54) | | - |
| *Non-EU* | | - | | 0.82 (0.55-1.24) | | - |
| **Local language competency** (Ref. no migration background) | | | | | | |
| *Poor/fair* | | - | | 0.53 (0.25-1.14) | | - |
| *Good/very good* | | - | | 1.23 (0.78-1.92) | | - |
| **Self-rated health** (Ref. Good) | | | | | | |
| *Poor* | | - | | - | | 0.89 (0.55-1.43) |
| **Health literacy** (Ref. High) | | | | | | |
| *Low* | | - | | - | | 0.71 (0.37-1.35) |
| **Trust in physicians (**Ref. *High)* | | | | | | |
| *Low* | | - | | - | | 1.88 (1.18-2.98)* |
| *Medium* | | - | | - | | 1.50 (0.98-2.29) |
| **Perceived discrimination (**Ref. *No)* | | | | | | |
| *Yes* |  | |  | | 1.53 (0.89-2.63) | |

Note: Sample characteristics were weighted by the age and gender distribution of the underlying population

*p<0.05;

^#^Sweden excluded from model 3 as information on health literacy and trust in physicians was not collected

Supplementary Table 5: Socio-demographic, migration-related and health-related factors associated with relying on the Internet for information when addressing health concerns (multivariable logistic regression): Birmingham, UK (n=524)

| **Variables** |  | | **Model 1**  **OR (95% CI)** | | **Model 2**  **OR (95% CI)** | | **Model 3**  **OR (95% CI)** |
| --- | --- | --- | --- | --- | --- | --- | --- |
| **Migration background** (Ref. no migration background) | | | | | | | |
| *Migrants* | | | 0.80 (0.42-1.50) | | - | | 0.76 (0.40-1.44) |
| *Descendants of migrants* | | | 0.95 (0.48-1.87) | | - | | 0.91 (0.46-1.80) |
| **Age in years** (Ref. 18-29) | | |  | |  | |  |
| *30-44* | | | 0.59 (0.34-1.03) | | 0.66 (0.37-1.17) | | 0.59 (0.34-1.04) |
| *45-59* | | | 0.22 (0.11-0.40)* | | 0.22 (0.12-0.41)* | | 0.23 (0.12-0.44)* |
| *≥60* | | | 0.12 (0.05-0.27)* | | 0.12 (0.06-0.28)* | | 0.14 (0.06-0.34)* |
| **Gender** (Ref. Women) | | | | | | | |
| *Men* | | | 0.73 (0.46-1.14) | | 0.74 (0.47-1.17) | | 0.75 (0.47-1.19) |
| **Education** (Ref. High) | | | | | | | |
| *Low* | | | 0.31 (0.18-0.54)* | | 0.37 (0.21-0.65)* | | 0.33 (0.19-0.59)* |
| *Medium* | | | 0.85 (0.47-1.53) | | 0.89 (0.49-1.62) | | 0.90 (0.50-1.64) |
| **Unemployed** (Ref. no) | | | | | | | |
| *Yes* | | | 0.48 (0.21-1.13) | | 0.48 (0.21-1.11) | | 0.45 (0.19-1.06) |
| **Region of origin** (Ref. no migration background) | | | | | | | |
| *EU-15* | | | - | | 1.11 (0.40-3.07) | | - |
| *EU-27* | | | - | | 0.98 (0.30-3.23) | | - |
| *Non-EU* | | | - | | 0.95 (0.48-1.85) | | - |
| **Local language competency** (Ref. no migration background) | | | | | | | |
| *Poor/fair* | | | - | | 0.31 (0.13-0.74)* | | - |
| *Good/very good* | | | - | | 1.05 (0.60-1.84) | | - |
| **Self-rated health** (Ref. Good) | | | | | | | |
| *Poor* | | | - | | - | | 0.78 (0.41-1.50) |
| **Health literacy** (Ref. High) | | | | | | | |
| *Low* | | | - | | - | | 0.85 (0.34-2.09) |
| **Trust in physicians (**Ref. High) | | | | | | | |
| *Low* | | | - | | - | | 1.60 (0.85-3.03) |
| *Medium* | | | - | | - | | 1.04 (0.62-1.73) |
| **Perceived discrimination (**Ref. No) | | | | | | | |
| *Yes* | |  | |  | | 1.15 (0.49-2.70) | |

Note: Sample characteristics were weighted by the age and gender distribution of the underlying population

*p<0.05

Supplementary Table 6: Socio-demographic, migration-related and health-related factors associated with relying on the Internet for information when addressing health concerns (multivariable logistic regression): Bremen, Germany (n=841)

| **Variables** |  | | **Model 1**  **OR (95% CI)** | | **Model 2**  **OR (95% CI)** | | **Model 3**  **OR (95% CI)** |
| --- | --- | --- | --- | --- | --- | --- | --- |
| **Migration background** (Ref. no migration background) | | | | | | | |
| *Migrants* | | | 0.63 (0.36-1.10) | | - | | 0.73 (0.41-1.31) |
| *Descendants of migrants* | | | 1.26 (0.82-1.94) | | - | | 1.16 (0.74-1.80) |
| **Age in years** (Ref. 18-29) | | |  | |  | |  |
| *30-44* | | | 1.11 (0.66-1.87) | | 1.13 (0.67-1.92) | | 1.01 (0.58-1.76) |
| *45-59* | | | 0.55 (0.34-0.89)* | | 0.57 (0.35-0.93)* | | 0.50 (0.30-0.83)* |
| *≥60* | | | 0.30 (0.19-0.47)* | | 0.32 (0.20-0.51)* | | 0.31 (0.19-0.51)* |
| **Gender** (Ref. Women) | | | | | | | |
| *Men* | | | 0.79 (0.57-1.10) | | 0.77 (0.55-1.08) | | 0.85 (0.60-1.20) |
| **Education** (Ref. High) | | | | | | | |
| *Low* | | | 0.33 (0.19-0.59)* | | 0.31 (0.17-0.56)* | | 0.30 (0.16-0.55)* |
| *Medium* | | | 0.62 (0.43-0.89)* | | 0.60 (0.41-0.86)* | | 0.63 (0.43-0.92)* |
| **Unemployed** (Ref. no) | | | | | | | |
| *Yes* | | | 0.91 (0.51-1.63) | | 0.95 (0.52-1.73) | | 0.94 (0.50-1.77) |
| **Region of origin** (Ref. no migration background) | | | | | | | |
| *EU-15* | | | - | | 0.49 (0.21-1.17) | | - |
| *EU-27* | | | - | | 1.36 (0.75-2.46) | | - |
| *Non-EU* | | | - | | 1.79 (0.99-3.22) | | - |
| **Local language competency** (Ref. no migration background) | | | | | | | |
| *Poor/fair* | | | - | | 0.24 (0.09-0.59)* | | - |
| *Good/very good* | | | - | | 0.56 (0.26-1.20) | | - |
| **Self-rated health** (Ref. Good) | | | | | | | |
| *Poor* | | | - | | - | | 0.84 (0.51-1.39) |
| **Health literacy** (Ref. High) | | | | | | | |
| *Low* | | | - | | - | | 0.95 (0.53-1.72) |
| **Trust in physicians (**Ref. High) | | | | | | | |
| *Low* | | | - | | - | | 2.81 (1.57-5.06)* |
| *Medium* | | | - | | - | | 2.10 (1.18-3.75)* |
| **Perceived discrimination (**Ref. No) | | | | | | | |
| *Yes* | |  | |  | | 1.06 (0.57-1.98) | |

Note: Sample characteristics were weighted by the age and gender distribution of the underlying population

*p<0.05

Supplementary Table 7: Socio-demographic, migration-related and health-related factors associated with relying on the Internet for information when addressing health concerns (multivariable logistic regression): Lisbon, Portugal (n=572)

| **Variables** | | **Model 1**  **OR (95% CI)** | | **Model 2**  **OR (95% CI)** | | **Model 3**  **OR (95% CI)** |
| --- | --- | --- | --- | --- | --- | --- |
| **Migration background** (Ref. no migration background) | | | | | | |
| *Migrants* | | 0.33 (0.13-0.82)* | | - | | 0.32 (0.10-1.02) |
| *Descendants of migrants* | | 1.20 (0.33-4.40) | | - | | 1.41 (0.37-5.47) |
| **Age in years** (Ref. 18-29) | |  | |  | |  |
| **30-44** | | 0.38 (0.17-0.87)* | | 0.38 (0.17-0.89)* | | 0.27 (0.10-0.70)* |
| **45-59** | | 0.29 (0.11-0.79)* | | 0.28 (0.10-0.78)* | | 0.29 (0.10-0.87)* |
| **≥60** | | 0.06 (0.02-0.21)* | | 0.06 (0.02-0.20)* | | 0.09 (0.02-0.37)* |
| **Gender** (Ref. Women) | | | | | | |
| *Men* | | 0.65 (0.33-1.28) | | 0.67 (0.34-1.32) | | 0.58 (0.27-1.23) |
| **Education** (Ref. High) | | | | | | |
| *Low* | | 0.77 (0.31-1.89) | | 0.77 (0.31-1.92) | | 0.63 (0.23-1.74) |
| *Medium* | | 0.66 (0.24-1.82) | | 0.57 (0.19-1.66) | | 0.52 (0.17-1.60) |
| **Unemployed** (Ref. no) | | | | | | |
| *Yes* | | 0.17 (0.03-0.83)* | | 0.15 (0.03-0.78)* | | 0.11 (0.02-0.74)* |
| **Region of origin** (Ref. no migration background) | | | | | | |
| *EU-15* | | - | | 1^#^ | | - |
| *EU-27* | | - | | 1^#^ | | - |
| *Non-EU* | | - | | 1.57 (0.37-6.56) | | - |
| **Local language competency** (Ref. no migration background) | | | | | | |
| *Poor/fair* | | - | | 0.10 (0.02-0.64)* | | - |
| *Good/very good* | | - | | 0.35 (0.07-1.67) | | - |
| **Self-rated health** (Ref. Good) | | | | | | |
| *Poor* | | - | | - | | 1.00 (0.38-2.61) |
| **Health literacy** (Ref. High) | | | | | | |
| *Low* | | - | | - | | 0.59 (0.18-1.94) |
| **Trust in physicians (**Ref. High) | | | | | | |
| *Low* | | - | | - | | 1.52 (0.41-5.71) |
| *Medium* | | - | | - | | 3.99 (1.78-9.08)* |
| **Perceived discrimination (**Ref. No) | | | | | | |
| *Yes* |  | |  | | 2.74 (0.74-10.15) | |

Note: Sample characteristics were weighted by the age and gender distribution of the underlying population

*p<0.05

^#^ total of 12 observations dropped as categories predicted outcome perfectly

Supplementary Table 8: Socio-demographic, migration-related and health-related factors associated with relying on the Internet for information when addressing health concerns (multivariable logistic regression): Uppsala, Sweden (n=571)

| **Variables** | **Model 1**  **OR (95% CI)** | **Model 2**  **OR (95% CI)** |
| --- | --- | --- |
| **Migration background** (Ref. no migration background) | | |
| *Migrants* | 0.84 (0.46-1.52) | - |
| *Descendants of migrants* | 1.05 (0.50-2.20) | - |
| **Age in years** (Ref. 18-29) | | |
| *30-44* | 1.83 (0.89-3.74) | 1.96 (0.93-4.11) |
| *45-59* | 0.78 (0.38-1.59) | 0.75 (0.35-1.59) |
| *≥60* | 0.62 (0.33-1.17) | 0.61 (0.32-1.18) |
| **Gender** (Ref. Women) | | |
| *Men* | 1.18 (0.75-1.85) | 1.20 (0.75-1.93) |
| **Education** (Ref. High) | | |
| *Low* | 0.25 (0.09-0.67)* | 0.23 (0.09-0.61)* |
| *Medium* | 0.66 (0.41-1.06) | 0.60 (0.37-0.99)* |
| **Unemployed** (Ref. no) | | |
| *Yes* | 0.58 (0.19-1.77) | 0.57 (0.18-1.82) |
| **Region of origin** (Ref. no migration background) | | |
| *EU-15* | - | 0.99 (0.45-2.19) |
| *EU-27* | - | 0.71 (0.19-2.73) |
| *Non-EU* | - | 1.18 (0.43-3.21) |
| **Local language competency** (Ref. no migration background) | | |
| *Poor/fair* | - | 0.08 (0.01-0.98)* |
| *Good/very good* | - | 0.93 (0.36-2.40) |

Note: Sample characteristics were weighted by the age and gender distribution of the underlying population

*p<0.05
